# The Role of p300 Histone Acetyltransferase in UV-Induced Histone Modifications and MMP-1 Gene Transcription

**DOI:** 10.1371/journal.pone.0004864

**Published:** 2009-03-16

**Authors:** Min-Kyoung Kim, Jung-Min Shin, Hee Chul Eun, Jin Ho Chung

**Affiliations:** 1 Department of Dermatology, Seoul National University College of Medicine, Seoul, Korea; 2 Laboratory of Cutaneous Aging Research, Clinical Research Institute, Seoul National University Hospital, Seoul, Korea; 3 Institute of Dermatological Science, Seoul National University, Seoul, Korea; University of Minnesota, United States of America

## Abstract

Matrix metalloproteinase (MMP)-1 promotes ultraviolet (UV)-triggered long-term detrimental effects such as cancer formation and premature skin aging. Although histone modifications may play a crucial role in the transcriptional regulation of MMP-1, the relationship between UV-induced histone modification and MMP-1 expression is not completely understood. Here, we identify regulators of histone acetylation that may link UV-mediated DNA damage and MMP-1 induction by UV in cultured human dermal fibroblasts (HDFs) *in vitro*. UV irradiation of HDFs induced MMP-1 expression and increased the level of phosphorylation of H2AX (γ-H2AX), p53 and the acetylation of histone H3 (acetyl-H3). Total histone deacetylase (HDAC) enzymatic activity was decreased by UV irradiation, while histone acetyltransferase (HAT) activity was increased. Suppression of p300 histone acetyltransferase (p300HAT) activity by the p300HAT inhibitor anacardic acid (AA) or by down-regulation of p300 by siRNA prevented UV-induced MMP-1 expression and inhibited UV-enhanced γ-H2AX, p53 level, and acetyl-H3. Using chromatin immunoprecipitation assays, we observed that γ-H2AX, p53, acetyl-H3, p300 and c-Jun were consistently recruited by UV to a distinct region (−2067/−1768) adjacent to the p300 binding site (−1858/−1845) in the MMP-1 promoter. In addition, these recruitments of γ-H2AX, p53, acetyl-H3, p300 and c-Jun to the p300-2 site were significantly abrogated by post-treatment with AA. Furthermore, overexpression of p300 increased the basal and UV-induced MMP-1 promoter activity. Our results suggest that p300HAT plays a critical role in the transcriptional regulation of MMP-1 by UV.

## Introduction

Matrix metalloproteinase (MMP)-1, commonly known as interstitial collagenase, is able to cleave interstitial collagens [1]. It belongs to a superfamily of zinc-dependent endopeptidases that are capable of degrading extracellular matrix components [2]. Its expression is correlated to connective tissue remodelling, e.g., tissue morphogenesis, wound healing, angiogenesis, tumor invasion, and metastasis [3], [4]. Indeed, excessive damage of connective tissue involves destruction of functional tissue structure, as in rheumatoid arthritis, osteoarthritis, autoimmune blistering disorders of skin, and dermal photoaging [5], [6], [7]. MMP-1 promotes ultraviolet (UV)-triggered long-term detrimental effects like cancer formation and premature skin aging [7], [8].

UV radiation is a major exogenous toxic agent [9] that leads to cellular damage and effects such as sunburn, immune suppression, skin cancer and photoaging [10]. The cellular response to DNA damage by UV radiation involves specific repair pathways such as nucleotide excision repair (NER). NER removes helix-distorting DNA lesions, including UV-induced cyclobutane pyrimidine dimers and 6–4 photoproducts [11], which are converted into more critical secondary lesions, e.g., DNA double-strand breaks (DSBs), through replication [12]. DSBs lead to activation of p53 protein, which rapidly co-localizes with γ-H2AX [13]. The stabilization and transactivation of p53 in response to DNA damage may be mediated through CBP/p300 [14] and/or PCAF [15], [16].

The transcriptional co-activator protein p300 acts as a key player in cellular differentiation and growth control and coordinates and integrates multiple signal-dependent processes at the transcriptional level through its histone acetyltransferase activity. This allows p300 to influence chromatin activity through histone modification and to modify transcription factors, gene transcription [17], protein-protein interactions, and protein stability. Hypoacetylation, on the other hand, which is induced by histone deacetylases, is associated with suppression of gene expression [12]. Although histone modifications may play a crucial role in the transcriptional regulation of MMP-1, the relationship between UV-induced DNA damage and histone modification is not completely understood.

Here, we identify regulators of histone acetylation that causally link UV-mediated DNA damage and induction of MMP-1 in primary human dermal fibroblasts (HDFs) *in vitro*. We show that total HDAC enzymatic activity was decreased and HAT activity was increased by UV irradiation. Suppression of p300HAT activity by anacardic acid (AA) [18], [19] or down-regulation of p300 via siRNA repressed UV-induced MMP-1 expression and inhibited UV-induced γ-H2AX, p53 level, and acetyl-H3. We also demonstrated that γ-H2AX, p53, p300, acetyl-H3 and c-Jun are recruited to a specific region (−2067/−1768) adjacent to the p300 binding site (−1858/−1845) in the MMP-1 promoter in response to UV irradiation; AA prevented this recruitment. Therefore, p300HAT plays a pivotal role in regulating chromatin remodeling events by UV radiation that lead to MMP-1 transcriptional activity.

## Results

### UV irradiation increased γ-H2AX, p53, acetyl-H3 levels, and MMP-1 expression in HDFs

**Figure 1:**
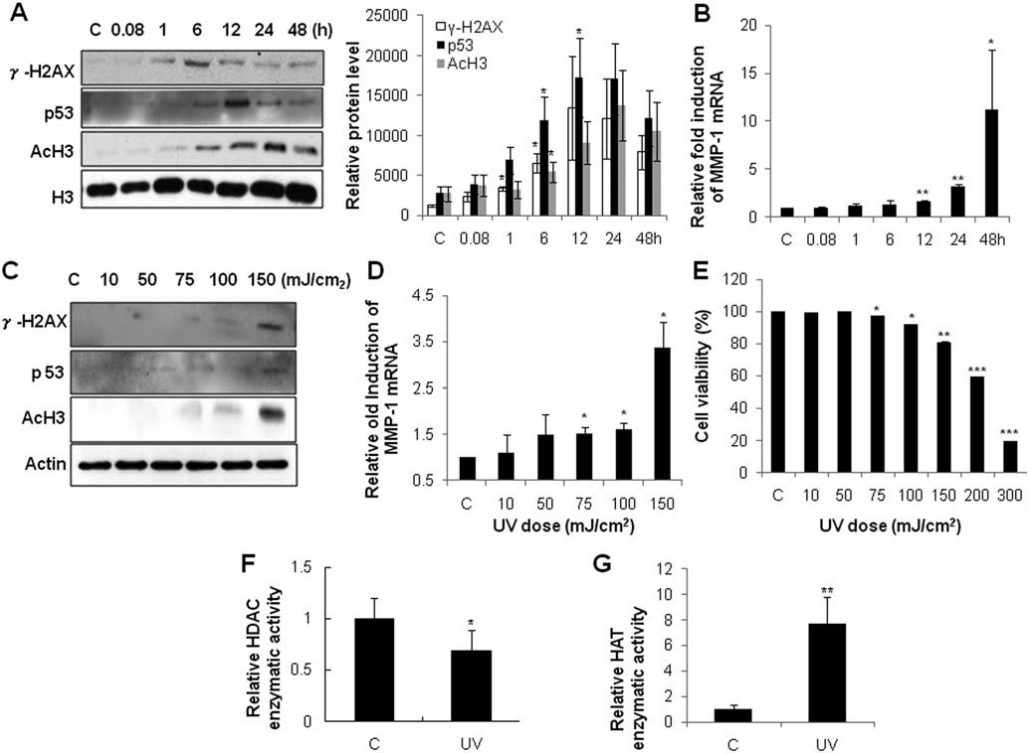
UV irradiation increased γ-H2AX, p53, acetyl-H3 levels and MMP-1 expression in HDFs. A, HDFs were UV-irradiated with 150 mJ/cm^2^ and cells were incubated for the indicated time. γ-H2AX, p53, and acetyl-H3 levels in total cell lysates were analyzed by western blotting (*left panels*). Bar graphs (*right panels*) show quantitative analysis of scanning densitometric values of γ-H2AX, p53 and acetyl-H3 as ratios to β-actin or histone H3, which were used as a loading control. Data shown are representative of four independent experiments. B, Time-dependent increases of MMP-1 mRNA expression following 150 mJ/cm^2^ of UV irradiation in HDFs. Expression of MMP-1 mRNA was detected by quantitative real-time RT-PCR and was normalized to the respective 36B4 mRNA. C, UV induced γ-H2AX, p53 level, acetyl-H3 in a dose-dependent manner (0–150 mJ/cm^2^). The levels of γ-H2AX, p53 and acetyl-H3 were measured by western blot analysis at 6 h after UV irradiation. D, MMP-1 mRNA (0–150 mJ/cm^2^) expression was quantified by real-time RT-PCR and was normalized to the respective 36B4 mRNA. E, Cytotoxic effects induced by increasing doses of UV irradiation (0–300 mJ/cm^2^) on HDFs measured by a MTT assay. F, Total cellular HDAC enzymatic activity was measured using an HDAC assay kit in untreated control HDFs or in HDFs UV-irradiated for 6 h. G, HAT activity in nuclear lysates of HDFs was determined after UV-irradiation with an indirect ELISA that detects acetyl residues. Values represent the mean±SEM of data from three independent experiments. C: control, UV: UV-irradiated cells, *P<0.05, **P<0.01, ***P<0.001 *vs.* control.

UV irradiation induces DNA damage and histone modification, which may activate various DNA repair genes, including p53 [20]. To investigate the effects of UV on γ-H2AX, a DNA DSB marker, p53, acetyl-H3, and MMP-1 expression, HDFs were treated with 150 mJ/cm^2^ of UV. We observed that γ-H2AX began to increase significantly at 1 h post-UV, and p53 and acetyl-H3 levels increased significantly from 6 h post-UV (Fig. 1A, p<0.05). MMP-1 mRNA expression was increased from 12 h post-UV in a time-dependent manner (Fig. 1B). Our results suggest that histone modification may precede the induction of MMP-1 mRNA expression. We also demonstrated the UV induced γ-H2AX, p53 level, acetyl-H3, and MMP-1 expression in a dose-dependent manner. We found that the relatively low UV doses of 75 mJ/cm^2^ and 100 mJ/cm^2^ induced γ-H2AX, p53 level, and acetyl-H3 slightly (Fig. 1C), and increased the MMP-1 mRNA expression by 1.5 and 1.6 folds, respectively (Fig. 1D). The HDFs were viable more than 80% by 150 mJ/cm^2^ after 48 hours post-UV (Fig. 1E).

Furthermore, HDAC activity was reduced by UV irradiation by 31.4±6.8% (p<0.05, Fig. 1F) at 6 h post-UV, while cellular HAT activity was increased by 776±53% (p<0.01, Fig. 1G). These observations suggested that UV induced the DNA damage and its repair gene, p53, and increased the acetylation of histone by decreasing HDAC and increasing HAT activities in HDFs.

### Histone deacetylase inhibitors induced MMP-1 expression

**Figure 2:**
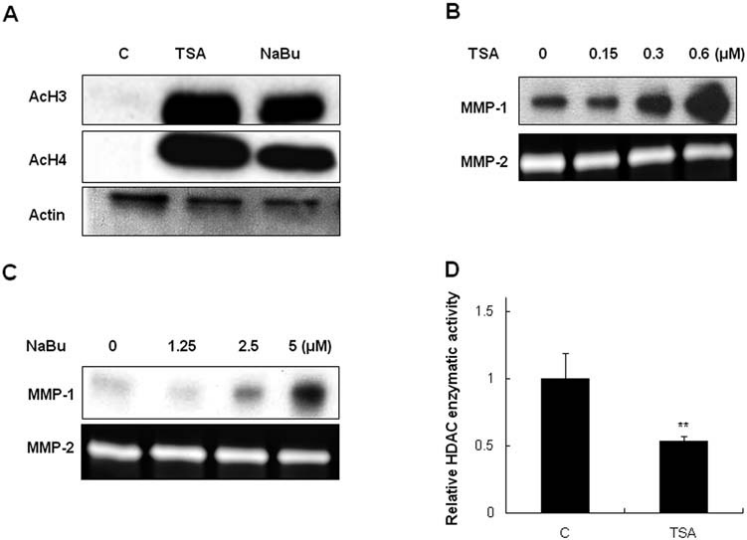
HDAC inhibitors induced MMP-1 expression. A, Western blot of cell lysates of HDFs after 6 h incubation with HDAC inhibitors, TSA and NaBu. Acetyl-H3 and acetyl-H4 were assessed using antibodies against acetyl-H3 and acetyl-H4. B, C, The amounts of MMP-1 and MMP-2 proteins released into culture media from HDFs were analyzed 24 h post-treatment with TSA or NaBu by western blotting (MMP-1) or zymography (MMP-2). D, Total cellular HDAC enzymatic activity was measured using a HDAC assay kit in untreated control HDFs or in HDFs treated with TSA for 6 h. Values represent the mean±SEM of data from three independent experiments. **P<0.01 *vs.* control.

Histone acetylation is regulated by the opposing actions of HAT enzymes and HDAC enzymes. To study the relationship between histone acetylation and MMP-1 expression in HDFs, we used two HDAC inhibitors, trichostatin A (TSA) and sodium butylate (NaBu). 0.6 µM TSA and 5 µM NaBu induced acetylation of histone H3 and H4 in HDFs after 6 h (Fig. 2A). Both TSA and NaBu increased MMP-1 expression in a dose-dependent manner after 24 h (Figs. 2B, C). On the other hand, MMP-2 protein levels were unaffected by TSA or NaBu treatment. As expected, total HDAC enzymatic activity was reduced in TSA-treated HDFs compared to that in control HDFs (46.5±13%, p<0.01, Fig. 2D). These results indicate that an increase in histone acetylation may contribute to the induction of MMP-1 expression.

### AA or p300 siRNA suppressed UV-induced MMP-1 expression and inhibited UV-enhanced levels of γ-H2AX, p53 and acetyl-H3

**Figure 3:**
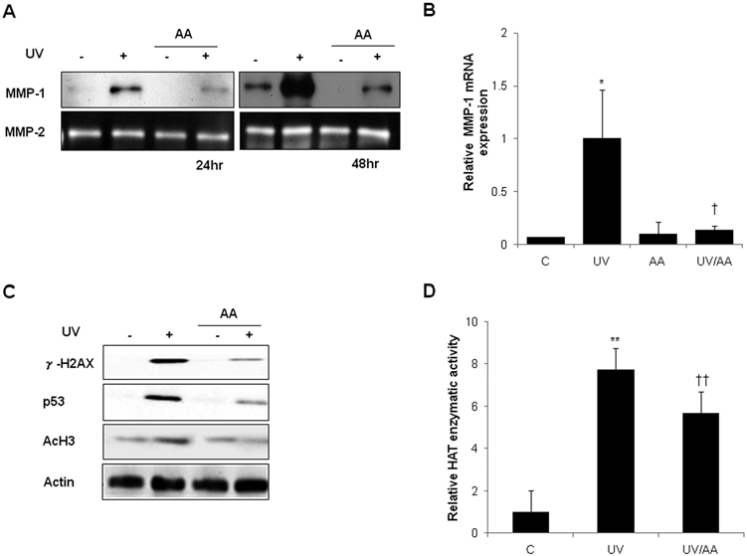
AA suppressed UV-induced MMP-1 expression and inhibited UV-enhanced levels of γ-H2AX, p53, and acetyl-H3. A, HDFs were incubated with AA for 24 h or 48 h after UV radiation and MMP-1 expression was analyzed by western blot. MMP-2 protein levels were analyzed by zymography. B, Level of MMP-1 mRNA was quantified by real-time RT-PCR and was normalized to the respective 36B4 mRNA levels. C, 6 h after 150 mJ/cm^2^ irradiation, HDFs with or without AA treatment were examined by western blot for γ-H2AX, p53 and acetyl-H3. D, HAT activity of nuclear lysates was determined in HDFs with or without AA treatment after UV-irradiation through an indirect ELISA that detects acetyl residues. C: control, UV: UV-irradiated cells, AA: AA treated cells, UV/AA: UV irradiated cells incubated with AA. *P<0.05, **P<0.01 *vs.* control; † P<0.05, † † P<0.01 *vs.* UV.

p300HAT is known to regulate transcription, chromatin structure, and DNA repair [21]. To investigate the role of p300HAT in UV-induced MMP-1 expression, HDFs were treated with 7.5 µM AA immediately after UV irradiation; AA is known to inhibit p300HAT activity [22], [23]. We demonstrated by western blot analysis that AA inhibited UV-induced MMP-1 protein expression at 24 h and 48 h post-UV (Fig. 3A). According to zymography, MMP-2 protein levels were not affected by either UV or AA treatment (Fig. 3A). The expression of MMP-1 mRNA was increased dramatically (p<0.05 *vs*. control cells) by UV irradiation and 7.5 µM AA significantly inhibited the UV-induced MMP-1 mRNA expression by 87±3.7% (p<0.05 *vs*. UV-irradiated cells, Fig. 3B). These data suggest that p300HAT may play a critical role in UV-induced MMP-1 expression. Furthermore, AA inhibited UV-enhanced level of γ-H2AX, p53, and acetyl-H3 at 6 h after UV (Fig. 3C), suggesting that p300HAT is involved in γ-H2AX, p53, and acetyl-H3 induction by UV. As expected, AA decreased the UV-induced HAT activity by 26±3.2% (p<0.001, Fig. 3D).

**Figure 4:**
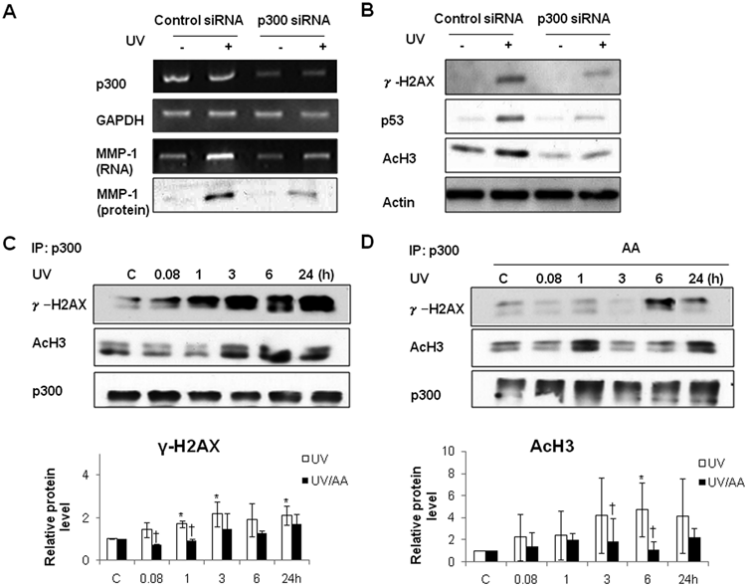
p300 siRNA suppressed UV-induced MMP-1 expression and inhibited UV-enhanced levels of γ-H2AX, p53 and acetyl-H3, and AA inhibited interaction of p300 with γ-H2AX or acetyl-H3 after UV. HDFs were transfected with scrambled control siRNA and p300 siRNA at 100 nM using Lipofectamine as recommended by the manufacturer. A, HDFs were irradiated with UV and incubated at 37°C 24 h after transfection with scrambled control or p300 siRNA. The MMP-1 mRNA level was analyzed by RT-PCR and the amount of MMP-1 protein was analyzed by western blot. B, The effect of p300 siRNA on the protein levels of γ-H2AX, p53, and acetyl-H3 were analyzed by western blotting. C, HDFs were UV-irradiated. After 6 h, whole cell lysates were immunoprecipitated with anti-p300 antibody. D, HDFs were UV-irradiated and post-treated with AA for 6 h. Whole cell lysates were immunoprecipitated with anti-p300 antibody. Proteins were immunoblotted with anti-p300, anti-acetyl-H3, and anti-γ-H2AX antibodies (*upper panels*). Bar graphs (*lower panels*) show quantitative analysis of scanning densitometric values of γ-H2AX, p53, and acetyl-H3 as ratios to β-actin, which was used as a loading control. Data shown are representative of three independent experiments. C: control, UV: UV-irradiated cells, AA: AA treated cells, UV/AA: UV irradiated cells incubated with AA. *P<0.05 *vs.* control, † P<0.05 *vs.* UV.

Next, we asked whether endogenous p300 had a similar impact on MMP-1 expression. To this end, we used siRNA to down-regulation of endogenous p300. Transient transfection of p300 siRNA in HDFs revealed that knockdown of p300 prevented UV-induced expression of MMP-1 mRNA and protein (Fig. 4A), indicating that p300 mediates UV-induced MMP-1 expression. Then, we investigated the roles of p300 in UV induction of γ-H2AX, p53, and acetyl-H3. Knockdown of p300 prevented UV induction of γ-H2AX, p53, and acetyl-H3 (Fig. 4B). These results confirmed that p300 plays an important role in UV-induced phosphorylation of H2AX, expression of p53 and acetylation of H3.

To further explore whether p300 interacts with γ-H2AX or acetyl-H3 and whether UV affects this interaction, immunoprecipitation with p300 antibody was performed using cell extracts prepared under the same conditions. We demonstrated that UV irradiation strongly increased the interaction of endogenous p300 with γ-H2AX and acetyl-H3 (Fig. 4C). The interaction of p300 with γ-H2AX increased from 1 h (p<0.05) post-UV, while the interaction with acetyl-H3 increased from 6 h post-UV (p<0.05). AA tended to inhibit UV-induced interaction of p300 with γ-H2AX or acetyl-H3 at all time points, and inhibited them significantly at 5 min–1 h (p<0.05) or 3–6 h (p<0.05) post-treatment, respectively (Fig. 4D). Taken together, our results suggest that p300 interacts with γ-H2AX or acetyl-H3 after UV and that the HAT activity of p300 may be involved in increasing γ-H2AX and acetyl-H3 after UV stimulation.

### p53 is involved in UV-induced acetylation of histone H3

**Figure 5:**
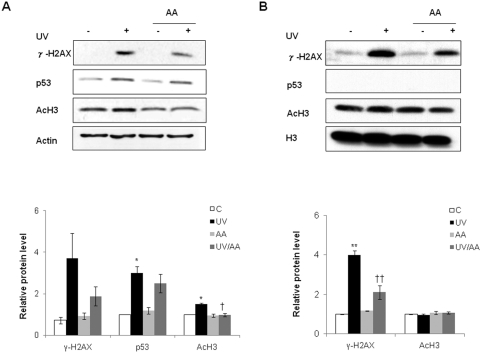
p300HAT mediates UV-induced p53 expression and histone modification. A and B, U2OS (p53^wt^) and SAOS-2 (p53^−/−^) cells were UV-irradiated and post-treated with 7.5 µM AA for 1 h. γ-H2AX, p53, and acetyl-H3 in total cell lysates were analyzed by western blotting (*upper panels*). Bar graphs (*lower panels*) show quantitative analysis of scanning densitometric values of γ-H2AX, p53 and acetyl-H3 as ratios to β-actin and histone H3 expression, which were used as loading controls. C: control, UV: UV-irradiated cells, AA: AA treated cells, UV/AA: UV irradiated cells incubated with AA. Values represent the mean±SEM of data from three independent experiments. *P<0.05, **P<0.01 *vs.* control; † P<0.05, † † P<0.01 *vs.* UV.

Because p53 is the primary mediator of UV-stimulated signaling, we investigated whether p53 is involved in the induction of γ-H2AX or acetyl-H3 by UV. U2OS (p53^wt^) and SAOS-2 (p53^−/−^) cells were irradiated with UV and then immediately treated with AA for 1 h. We demonstrated that UV induced γ-H2AX in both U2OS (Fig. 5A) and SAOS-2 cells (Fig. 5B). Furthermore, AA inhibited UV induction of γ-H2AX in both U2OS and SAOS-2 cells. On the other hand, UV enhanced acetyl-H3 in U2OS (Fig. 5A), but not in SAOS-2 (Fig. 5B), suggesting that acetylation of histone H3 is required for activation of the p53 signaling pathway. AA prevented UV induction of acetyl-H3 in U2OS cells, indicating that p300HAT activity plays a role in UV enhancement of acetyl-H3 in the presence of p53 (Fig. 5A). Taken together, these results indicate that acetylation of histone H3 depends on activation of p300HAT and p53.

### Recruitment of γ-H2AX, p53, p300, acetyl-H3, and c-Jun to a specific region (−2067/−1768) adjacent to the p300-2 binding site (−1858/−1845) in the MMP-1 promoter was increased after UV irradiation and AA prevented this recruitment

**Figure 6:**
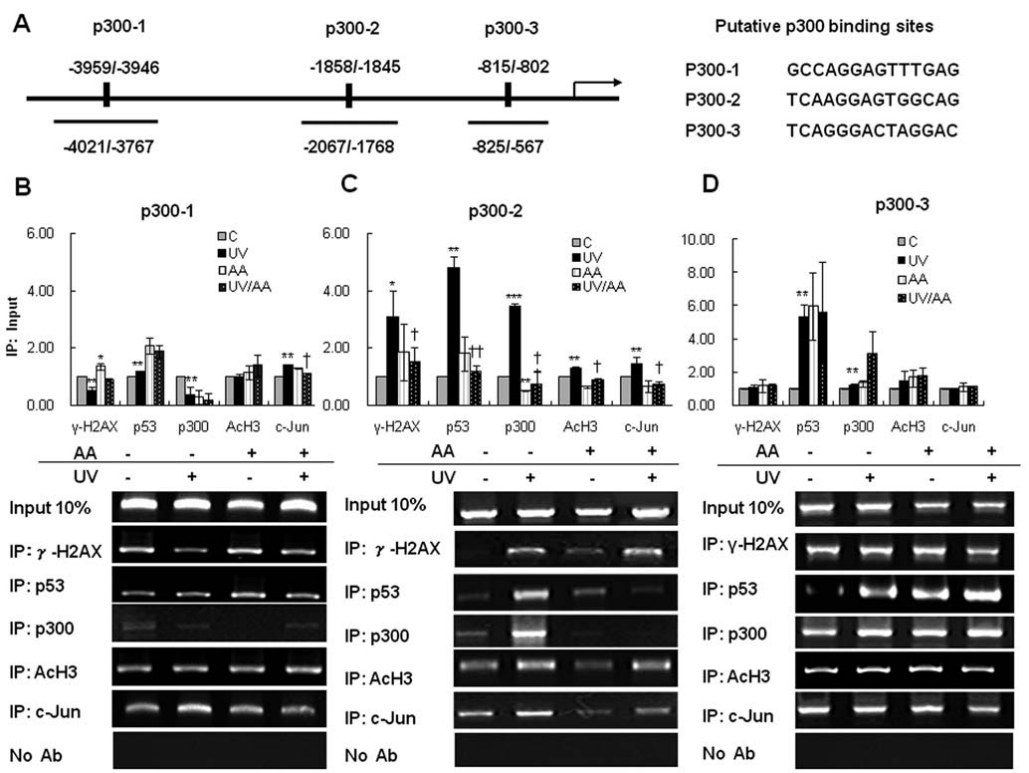
Regulatory elements in the human MMP-1 promoter region. A, Schematic of the MMP-1 promoter. The numbers indicate nucleotide positions relative to the translation start site. Putative p300 binding sites (p300-1, p300-2, p300-3) in the MMP-1 promoter are indicated. HDFs were UV-irradiated and post-treated with 7.5 µM AA for 6 h. HDFs were treated with 37% formaldehyde to cross-link proteins to DNA. The DNA fragments were immunoprecipitated using anti-γ-H2AX, anti-p53, anti-p300, anti-acetyl-H3 or anti-c-Jun antibodies, and MMP-1 promoter fragments were detected using PCR. PCR was performed to amplify the regions between −4021/−3767 for p300-1 (B), −2067/−1768 for p300-2 (C) and −825/−567 for p300-3 (D) of the MMP-1 gene. Band intensity obtained after IP was normalized to input control to show relative recruitment of γ-H2AX, p53, p300, acetyl-H3 or c-Jun to the MMP-1 promoter in HDFs (*lower panels*). Bar graphs (*upper panels*) show quantitative analyses of scanning densitometric values. Values are normalized by input DNA. PCR bands are representative of three separate chromatin immunoprecipitations. Results are expressed as the mean±SEM fold-induction vs. basal levels. C: control, UV: UV-irradiated cells, AA: AA treated cells, UV/AA: UV irradiated cells incubated with AA. *P<0.05, **P<0.01 ***P<0.001 *vs.* control. † P<0.05, †† P<0.01 *vs.* UV. ‡P<0.05, ‡‡ P<0.01 *vs.* control.

To determine whether UV induction of γ-H2AX, p53, and acetyl-H3 is directly involved in the transcriptional regulation of MMP-1 and to elucidate the role of p300HAT activity in MMP-1 transcription, we performed ChIP assays with antibodies specific to γ-H2AX, p53, p300, acetyl-H3, and c-Jun with or without AA treatment. Analysis of the MMP-1 promoter using TESS (http://www.cbil.upenn.edu/cgi-bin/tess/tess), and Motif (http://motif.genome.jp) revealed three putative p300 binding sites (p300-1, −3959/−3946; p300-2, −1858/−1845; p300-3, −815/−802, Fig. 6A). Primer pairs that would result in PCR products ranging from 180 to 300 bp were designed to amplify the DNA corresponding to the above sites, and were used to measure the immunoprecipitated DNA fragments by PCR. The recruitment of γ-H2AX was increased at only the p300-2 site of MMP-1 promoter following UV irradiation, but was decreased at the p300-1 site and unchanged at the p300-3 site. AA prevented UV induction of γ-H2AX at the p300-2 site, but did not affect γ-H2AX at the other two sites.

The binding of p53 to the p300-1, p300-2 and p300-3 sites of the MMP-1 promoter was significantly increased (p<0.001, Fig. 6B; p<0.01, Fig. 6C; p<0.01, Fig. 6D) at 6 h post-UV. However, AA prevented UV-induced binding of p53 at only the p300-2 site (p<0.05, Fig. 6C), but UV-induced binding of p53 at the p300-1 and p300-3 sites tended to increase and remain unchanged by AA treatment, respectively. The p53 binding element was found in the regions surrounding all three p300 binding sites of the MMP-1 promoter.

At 6 h post-UV stimulation, p300 was recruited to the p300-2 site (p<0.001, Fig. 6C) and p300-3 site (p<0.01, Fig. 6D), while p300 recruitment to the p300-1 site was decreased after UV stimulation (p<0.05, Fig. 6B). AA prevented UV-induced recruitment of p300 to only the p300-2 site (p<0.01). In contrast to the p300-2 site, AA tended to augment UV-induced recruitment of p300 to the p300-3 site. In addition, the acetyl-H3 level was significantly increased (p<0.01, Fig. 6C) at only the p300-2 site, but did not change at the p300-1 or p300-3 site following UV irradiation. AA significantly prevented UV-induced acetyl-H3 at only the p300-2 site (p<0.05, Fig. 6C), but did not affect acetyl-H3 at the other two sites.

Our results indicate that γ-H2AX, p53, p300, and acetyl-H3 were consistently recruited by UV irradiation to a distinct region (−2067/−1768) near the p300-2 binding site, whereas their recruitment to the p300-1 and p300-3 sites was variable (Figs. 6B, D). In addition, the recruitments of γ-H2AX, p53, p300 and acetyl-H3 to the −2067/−1768 sequence around the p300-2 site were significantly abrogated by post-treatment with 7.5 µM AA (Fig. 6C), confirming that the p300-2 site is one of the most important regions in the MMP-1 promoter in terms of MMP-1 transcriptional regulation after UV irradiation.

Next we asked whether the transcriptional induction of MMP-1 is associated with c-Jun at the MMP-1 promoter. The transcriptional activity of MMP-1 is known to be mediated by AP-1 and the MAPK pathway. AP-1 binding element exists in the p300-2, p300-3 in the MMP-1 promoter. We demonstrated significant increases in c-Jun at −4021/−3767 around the p300-1 binding site, at −2067/−1768 around the p300-2 binding site, at 6 h post-UV irradiation (p<0.001, p<0.05). Furthermore, the binding of c-Jun to the p300-1 and p300-2 sites was decreased by AA treatment (p<0.01, p<0.01, Figs. 6C, D). In contrast, the binding affinity of c-Jun to the p300-3 site was not altered by either UV or AA (Fig. 6D). Taken together, these results support the conclusion that the distinct region (−2067/−1768) near the p300-2 binding site of the MMP-1 promoter is important for MMP-1 transcriptional activation by UV.

### p300 overexpression increased the basal and UV-induced MMP-1 promoter activity

**Figure 7:**
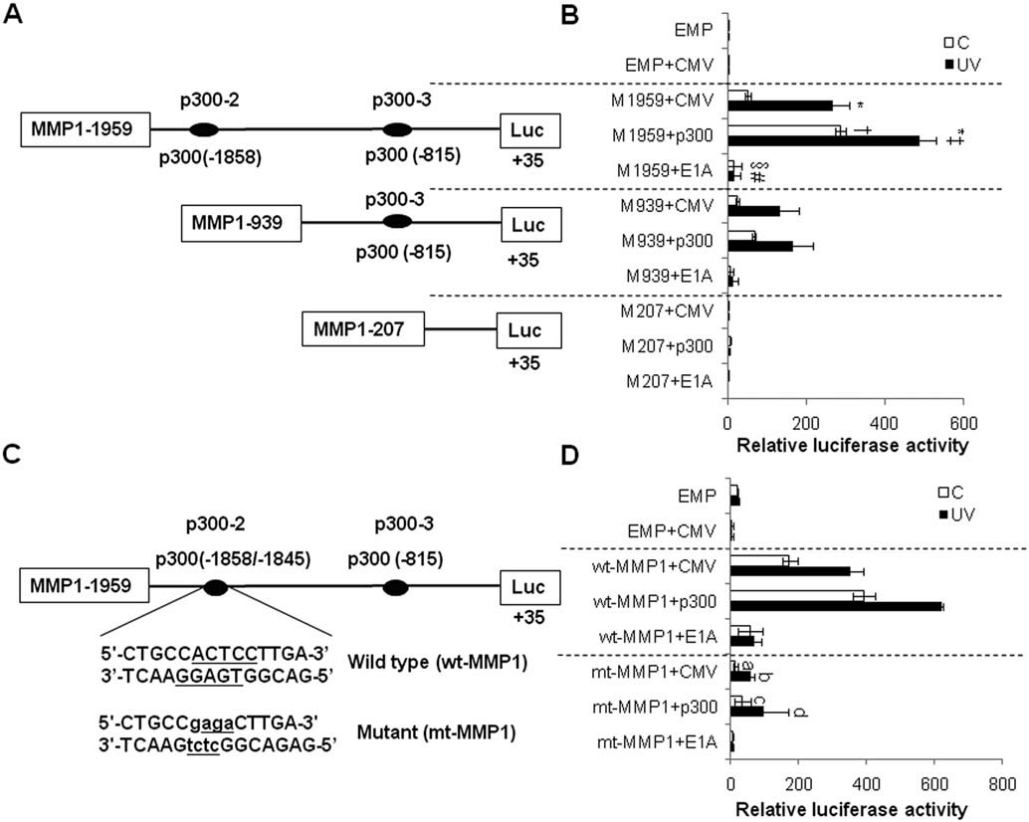
p300 overexpression increased MMP-1 promoter activity. A, Schematic structure of MMP-1 promoter constructs. B, HDFs were transiently transfected with a series of 5′ deletion hMMP1/luciferase reporter plasmids (MMP1-1959luc, MMP1-939luc, and MMP1-207luc) together with 200 ng of pCMV-wt-p300 plasmid or pCMV and pCMV-E1A as controls. The cells were subsequently UV-irradiated and incubated for 6 h. Cells were harvested and protein extracts were assayed for luciferase activity. Three independent transfections, each run in triplicate, were performed, and the results are expressed as the means±S.E.M. EMP, pGL3 vector; p300, p300 expression vector; E1A, E1A expression vector; * represent p<0.05 UV untreated cells. † represent p<0.05 *vs.* CMV with MMP-1. ‡ represent p<0.05 *vs.* CMV with MMP-1 in UV-untreated cells. § represent p<0.05 *vs.* p300 with MMP-1. # represent p<0.05 *vs.* p300 with MMP-1 in UV untreated cells. C, A sequences for putative p300-2 binding site (−1858/−1845). D, Effects of wild type and mutant p300-2 binding site on the promoter activity of the MMP-1 gene. The HDFs were co-transfected with MMP1-1959luc plasmid containing either wild type p300-2 binding site (wt-MMP1) or mutated p300-2 binding site of the MMP1 gene (mt-MMP1) with pCMV-wt-p300 plasmid or pCMV and pCMV-E1A as controls. Three independent transfections, each runs in triplicate, were performed, and the results are expressed as the means±S.E.M. EMP, pGL3 vector; p300, p300 expression vector; E1A, E1A expression vector; a represent p<0.05 *vs* CMV with wt-MMP1 in UV-untreated cells. b represent p<0.05 *vs.* CMV with wt-MMP1 in UV-treated cells. c represent p<0.05 *vs.* p300 with wt-MMP1 in UV-untreated cells. d represent p<0.05 *vs.* p300 with wt-MMP1 in UV-treated cells.

To investigate whether p300 overexpression can stimulate MMP-1 promoter activity and whether the p300-2 binding site is critical for MMP-1 transcription, HDFs were transiently co-transfected with various human MMP-1 luciferase reporter constructs (MMP1-1959luc, MMP1-939luc, MMP1-207luc) and a p300 or EIA construct (Fig. 7A). The E1A construct expresses p300 protein with functionally inactivated histone acetyltransferase activity [24]. The MMP1-1959luc construct includes the p300-2 binding site, which was essential for UV-induced increase of MMP-1 promoter activity as described above. The activity of MMP-1-1959luc promoter alone was significantly increased by UV (p<0.05 *vs* UV-untreated cells). Indeed, the basal and UV-induced MMP1-1959luc promoter activity was actually further enhanced in the presence of p300 (p<0.05 *vs* cells transfected with MMP1-1959luc alone). However, E1A dramatically reduced the basal and UV-induced MMP1-1959luc promoter activity by 96±1.1% and 81.1±2.1% (p<0.05 *vs*. cells transfected with p300, Fig. 7B). These results suggest that the p300-2 binding site is necessary for the basal and UV-induced transcriptional regulation of MMP-1. We also demonstrated that deletion of the p300-2 binding site (MMP1-939luc) abrogated the p300 overexpression-induced increase of basal and UV-induced MMP-1 promoter activity and that deletion of the p300-3 binding site (MMP1-207luc) completely abrogated the p300 overexpression-induced increase of MMP-1 promoter activity.

To confirm the importance of p300-2 binding site in the promoter region of the MMP-1 gene, GGAGT (−1853/−1849) in the consensus sequence of putative p300 binding site in MMP1-1959luc was mutated to Gtctc designated as mt-MMP1-1959luc (Fig. 7C). We demonstrated that mutation of the p300-2 binding site inhibited the p300 overexpression-induced increase of basal and UV-induced MMP-1 promoter activity completely (Fig. 7D). These results confirm that the p300-2 binding site is critical for the basal and UV-induced transcriptional regulation of MMP-1.

## Discussion

The present study demonstrated that p300HAT mediates the UV induction of MMP-1 expression in HDFs. We demonstrated that UV irradiation increased the acetylation of histone H3 by decreasing HDAC enzymatic activity and increasing HAT activity. Furthermore, the p300HAT inhibitor, AA, and knockdown of p300 by siRNA inhibited UV-induced acetylation of histone H3 and MMP-1 expression. Several transcriptional co-activators with intrinsic acetyltransferase activity have been identified, including PCAF, GCN5, CBP and p300 [25], [26] and AA was reported to inhibit p300-associated histone acetyltransferase activity [18]. AA has been shown to inhibit TNF-induced COX-2 and MMP-9 expression [27], which are induced also by UV irradiation in human skin *in vivo* [8].

Our study indicated that UV irradiation stimulated the HAT activity of p300 and led to increased histone acetylation, thereby relaxing chromatin structure and promoting MMP-1 activation. Interestingly, we also observed that UV induction of γ-H2AX and the expression of p53 was inhibited by AA or by knockdown of endogenous p300, indicating that p300HAT is also involved in UV induction of γ-H2AX and p53. UV irradiation also resulted in increased interaction of p300 protein with γ-H2AX or acetyl-H3. UV-induced DNA lesions led to an increase of γ-H2AX and p53 [28], and p300 stabilized the p53 for DNA repair [29]. Recently, evidence has been accumulating for the critical role of histone acetylation in the p300/p53-mediated transactivation of target genes by UV in the DNA repair process. There are several reports that DNA becomes more accessible during UV-induced DNA repair [30] and that histone acetylation stimulates the initial rate of NER *in vivo* by increasing the chromatin-enhanced DNA repair process that occurs early after UV irradiation [31]. We demonstrated that UV did not induce acetylation of histone H3 in the absence of p53, indicating that p53 plays an important role in histone acetylation by UV. It has been reported that p53 plays a part in the regulation of acetyl-H3 [32]. Furthermore, acetyl-H3 may require endogenous p300 and p53 complex [33], and p300 is a key regulator of the p53 response [34], [35].

Using ChIP assays, we demonstrated that UV irradiation increased the recruitment of γ-H2AX, p53, p300, acetyl-H3, and c-Jun to a specific region (−2067 to −1768) around the p300-2 binding site (−1858/−1845) in the MMP-1 promoter and that AA prevented these UV-induced recruitments. However, in contrast to the region around p300-2, we did not find increased recruitment of γ-H2AX, a known marker for DSB, in either the −4021/−3767 around the p300-1 binding site or the −825 to −567 region around the p300-3 binding site by UV irradiation. There are two possible explanations for these differences. First, these two regions may be less susceptible to DSB by UV and, thus, DSB would not occur in these two regions for unknown reasons. Second, DSB in these two regions may have already been repaired at this time point (6 h post-UV). Since we found significantly increased recruitment of p53 in these two regions, the second explanation may be more appropriate. Although we still do not understand the exact time-dependent sequence for DSB and its repair in the promoter regions of MMP-1 genes, we speculate that the −2067 to −1768 region around the p300-2 binding site (−1858/−1845) in the MMP-1 promoter may be involved in DNA repair processes at this time point and that p300 plays important roles in the DNA repair processes and in MMP-1 transcriptional regulation.

**Figure 8:**
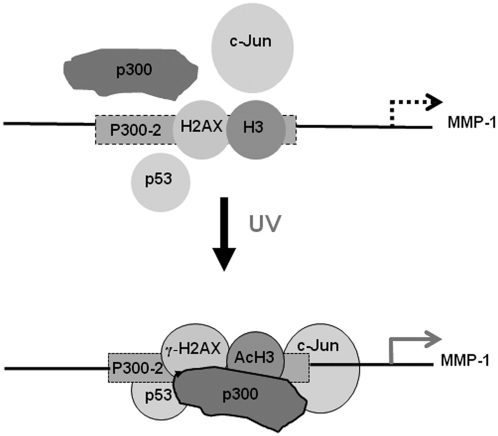
Scheme of γ-H2AX, AcH3, p53, and c-Jun interactions with p300 on the MMP-1 promoter after UV-irradiation. Transcriptional regulation of the MMP-1 gene by UV depends on the ordered coordination of γ-H2AX (DSB), recruitment of p300 and p53, hyperacetylation of histone, and increased binding of c-Jun on the MMP-1 promoter of putative p300-2 binding site.

p300 is reported to functionally collaborate with c-Jun on the MMP-1 promoter [36], and binding of p300 to the −1978/−1523 site of the MMP-1 promoter is redox-sensitive [37], which region is partially overlapped with −2067 to −1768 region around the p300-2 binding site of our study. Nevertheless, the mechanism of p300, role in UV-induced histone modification and UV-induced MMP-1 expression are still unclear. Our observations suggest that transcriptional regulation of the MMP-1 gene by UV depends on the ordered coordination of γ-H2AX (DSB), recruitment of p300 and p53, hyperacetylation of histone, and increased binding of c-Jun on the MMP-1 promoter (Fig. 8). Our results suggest that p300HAT-medated histone modification by UV is important in the transactivation of the MMP-1 gene. Overexpression of p300 in the presence of the p300-2 binding site led to dramatic increases of basal and UV-induced promoter activities of MMP-1, indicating that this specific region around p300-2 in the MMP-1 promoter may be critical for basal and UV-induced MMP-1 expression. Furthermore, our results demonstrate a novel role for AA in preventing UV-induced MMP-1 expression, suggesting that p300 inhibitors such as AA could be used for anti-skin aging cosmetics or drugs.

## Materials and Methods

### Cell culture and UV irradiation

Primary human dermal fibroblasts (HDFs), which were isolated from foreskin by outgrowth from biopsies of healthy donors of 7–30 y, and U2OS (p53+/+) and Saos-2 (p53−/−), which were derived from human osteosarcoma, were cultured in Dulbecco's modified Eagle's media (DMEM, Gibco, Rockville, MD) with glutamine (2 mM), penicillin (400 U/mL, Gibco, Rockville, MD), streptomycin (50 mg/mL, Gibco, Rockville, MD), and 10% FBS (Hyclone, Logan, USA) in a humidified 5% CO_2_ atmosphere at 37°C. Cultured HDFs at passages 6–10 were used for the experiments. Cells at 95% confluence were UV-irradiated by using a TL20W/12RS UV lamps (Philips, Eindhoven, Netherlands) with an emission spectrum between 275 and 380 nm (peak, 310–315 nm) [38]. The power output distribution of the UV emission spectrum was 10.2% UVC (275–290 nm), 53.3% UVB (290–320 nm), 25.3% UVA1 (320–340 nm) and 11.2% UVA2 (340–380 nm). A Kodacel filter (TA401/407; Kodak, Rochester, NY) was mounted 2 cm in front of the UV lamps to remove UVC (<290 nm) wavelengths. Irradiation intensity measured by using an UV meter (Model 585100; Waldmann Co., Villingen-Schwenningen, Germany) at a distance of 40 cm was 0.48–0.55 mW/cm^2^ [39].

After irradiation, DMEM with 10% FBS was added to the cells. The cells were then incubated for various time periods dependent on the experimental settings. Inhibitors were added to the cells after UV irradiation. Trichostatin A (Sigma, St. Louis) and sodium butylate (Sigma, St. Louis) were used as HDAC inhibitors and AA (Calbiochem, CA) was used as a p300HAT inhibitor. All human cells were cultured from the foreskin specimens obtained by circumcision, with prior informed verbal consents of participants. This study was approved by the institutional review boards of Seoul National University Hospital and conducted according to the Declaration of Helsinki principles.

### Cytotoxicity assay

The 3-(4,5-dimethylthiazol-2-yl)-2,5-diphenyltetrazoliumbromide (MTT) assay was used to evaluate the cell viability after exposure to UV irradiation (0, 10, 50, 75, 100, 150, 200, and 300 mJ/cm^2^). After irradiation the HDFs were incubated at 37°C for 48 h. Then, 50 ul of a MTT solution at 5 mg/ml in PBS was added, and the cells were incubated at 37°C for 4 h. In the last step, 500 ul of dimethylsulfoxide was added to each dish. Absorbance of the supernatant was measured at 540 nm against a background at 700 nm.

### Quantitative real time-PCR

Total RNA was isolated from HDFs using a Trizol (Invitrogen, Carlsbad, CA) and 1 µg of total RNA was converted to cDNA using First Strand cDNA Synthesis Kit (MBI Fermentas, Vilnius, Lithuania) according to the manufacturer's instructions. To quantitatively estimate the mRNA expression of MMP-1 genes, PCR was performed on a 7500 Real-time PCR System (Applied Biosystems, Foster City, CA) using the SYBR®Premix Ex Taq™ (Takara Bio Inc., Shiga, Japan) according to the manufacturer's instructions. Primers for MMP-1 are forward (5′-AAGCGTGTGACAGTAAGCTA-3′) and reverse (3′-AACCGGACTTCATCTCTG-5′). Primers for 36B4 are forward (5′-TGGGCTCCAAGCAGA TGC-3′) and reverse (3′-GGCTTCGCTGGCTCCCAC-5′). The PCR conditions were 50°C for 2 minutes, 95°C for 2 minutes, followed by 40 cycles at 95°C for 15 s and 60°C for 1 minute. Data were analyzed using the 2^−ΔΔCT^ methods [40], the data being presented as the fold in gene expression normalized to 36B4 and relative to the UV irradiated or control cells. These experiments were carried out in triplicate and independently repeated at least three times.

### Preparation of histone extracts

For the analysis of histone protein, cells were lysed with acidic buffer (10 mM HEPES, 1.5 mM MgCl_2_, 10 mM KCl, 0.4 mM DTT, 1.5 mM PMSF, 0.2 N HCl) for 30 min on ice and then centrifuged. The acid-soluble protein supernatant was dialyzed twice against 0.1 M acetic acid and three times against H_2_O for 1 h each. The supernatant was concentrated using an ultra filter column (Vivascience, Hamburg, Germany), and the protein was eluted in H_2_O.

### Immunoblotting, immunoprecipitation, and zymography

Protein extracts (20 µg) from treated HDFs were run on 10% to 15% SDS-PAGE, transferred to nitrocellulose membrane, and blotted with the following antibodies: rabbit anti-acetyl H3, anti-H3, anti-acetyl H4, anti-H4, anti-phospho-H2AX-ser139, and anti-H2A polyclonal antibodies (Upstate Biotech, NY) at a 1∶1000 dilution. Antibodies against p300 (Upstate Biotech, NY) and p53 (DO-1; Santa Cruz Biotechnology, CA) were also used. Immunoreactive proteins were visualized by enhanced chemiluminescence (Amersham, Buckinghamshire, UK). Immunoprecipitation assays were performed by binding the antibodies to 20 µl protein A/G-sepharose beads (Santa Cruz Biotechnology, CA). Subsequently, 500 µg of cell lysates were incubated with p300 antibody-coupled beads for 24 h at 4°C. Samples were washed and separated on 5% to 15% gel by SDS-PAGE. To determine the amount of MMP-1 secreted into culture media, equal aliquots of conditioned culture media from an equal number of HDFs were fractionated by 10% SDS-PAGE, transferred to Hybond ECL membrane (Amersham Biosciences, Buckinghamshire, England), and analyzed by western blotting with a rabbit polyclonal antibody against MMP-1. MMP-2 protein secretion and activation in the culture supernatants were measured using gelatin zymography under non-reducing conditions as described previously [41].

### Histone deacetylase activity assay

Total cellular HDAC enzymatic activity was measured using an HDAC assay kit (Upstate Biotech, NY) according to the manufacturer's protocol. Briefly, 20 µg of total cell extracts from HDFs were incubated with a fluorometric substrate in HDAC assay buffer for 20 min at 37°C. Activator solution was then added to release the fluorophore from the deacetylated substrates, and the fluorescence was measured in a fluorescence plate reader.

### Histone acetyltransferase assay

HAT activity was measured using a commercially available, non-radioactive HAT activity assay kit according to the detailed instructions provided by the manufacturer (Upstate Biotech, NY). HDFs lysates were incubated in 100 µl of HAT assay buffer supplemented with acetyl-CoA (100 µM) and biotinylated histone H3 peptide (0.5 µg) for 30 min at 30°C. Aliquots of the reactions were immobilized on streptavidin plates, and acetylation was detected using the HAT ELISA as described in the Upstate protocol [42].

### Chromatin immunoprecipitation Assay

Confluent HDFs were UV- or mock-irradiated with verification at various times after UV exposure. Genomic DNA and proteins were cross-linked at 37°C by formaldehyde (1% final concentration). The cells were scraped into PBS with 1 mM PMSF and pelleted at 2000 rpm for 4 min at 4°C. 200 µl of warm SDS lysis buffer containing protease inhibitors and 1 mM PMSF were then added and incubated for 10 min on ice. Cells were sonicated to generate 100- to 1000-bp DNA fragments. Before addition of antibody, 200 µl of lysate was saved as the input sample. Either 5 µg of anti-acetyl-H3, anti-phospho-H2AX-ser139, p300 (Upstate Biotech. NY), p53 (Santa Cruz, Biotechnology, CA, USA) or c-Jun (Cell Signaling, Beverly, MA) antibodies were added to the lysate with cold 1× PBS. Protein-bound, immunoprecipitated DNA was washed with LiCl wash buffer, 10 mM Tris and 1 mM EDTA (pH 8.0), and immune complexes were eluted with elution buffer (1% SDS and 0.1 M NaHCO_3_), followed by incubation for 4 h at 65°C in 20 µl 5 M NaCl to reverse the cross-linking and incubation for 1 h at 45°C with 10 µl of 0.5 M EDTA, 20 µl 1 M Tris HCl and 1 µl of 20 mg/ml proteinase K (Sigma-Aldrich, Steinheim, Germany). DNA was extracted with phenol/chloroform (Sigma-Aldrich, Steinheim, Germany), precipitated with isopropanol (Merck, Darmstadt, Germany), and resuspended in 20 µl of nuclease-free water. PCR was performed with specific primers to amplify regions between −4021/−3767 (p300-1), −2067/−1768 (p300-2) and −825/−567(p300-3) of the MMP-1 promoter. The sequences for all primers used in PCR for ChIP analysis were: p300-1 forward, 5′- TGT GGT GGC TCA CAC CTA-3′; p300-1 reverse, 3′- TCT GTC GCT TAG GCT GGA-5′; p300-2 forward, 5′-ACA TTG CAG GAT GTG CA-3′; p300-2 reverse, 3′-TGA CTC CAG CAC AGA CA-5′; p300-3 forward, 5′-AAC TTC AGT CAG TAC AGG-3′; p300-3 reverse, 3′-CCA GTG CTA TCT GTA GCA-5′.

### RNA interference

Endogenous p300 in HDFs was depleted through two sequential transfections. A total of 100 nM p300 siRNA, 100 nM scrambled control siRNA or 100 nM GAPDH siRNA were transfected into HDFs using Lipofectamine 2000 (Invitrogen, Carlsbad, CA, USA). After 24 h of transfection, cells were UV-irradiated and then harvested after 6 h and 24 h.

### Point Mutations of the p300-2 binding site in the promoter of the MMP-1 Gene

Site-directed mutagenesis was performed mainly according to the PCR-based mutagenesis kit (Quik Change site-directed mutagenesis kit, Stratagene, USA). The fragment of MMP-1 gene −2067/−1768 (p300-2) containing a putative p300 binding site (5′-CTGCCACTCCTTGA-3′) and (3′-TCAAGGAGTGGCAG-5′) was inserted into pGL3 (MMP1-1959luc). The consensus sequence GGAGT (−1853/−1849) of the p300 was mutated to Gtctc utilizing a mutagenic forward primer (5′-CACAGTCGAGTATA TCTGCCgagaCTTGACTTTTAAAACATAGTC-3′) and a reverse primer (3′-GACTA TGTTTTAAAAGTCAAGtctcGGCAGATATACTCGACTGTG-5′). The mutated sequences had been confirmed by DNA sequencing. For MMP-promoter activity assay, the DNA fragment (−1853/−1849) containing mutated p300 binding site (mt-MMP1-1959luc) was inserted into pGL3 basic vector.

### Plasmid constructs, transient transfection, and luciferase reporter assay

The human MMP-1 promoter/luciferase plasmids (MMP-1959luc, MMP1-939luc, MMP1-207luc, mt-MMP1-1959luc and wt-MMP1-1959luc) contained the firefly luciferase gene under the transcriptional control of the human MMP-1 promoter in the pGL3 basic reporter vector (Promega, Madison, WI, USA). For luciferase assays, HDFs were cultured in 12-well plates for 2 days before transfection. pCMV-E1A, pCMV-p300, and MMP1-Luc were transiently co-transfected into cells using Lipofectamine 2000 (Invitrogen, Carlsbad, CA, USA). At 24-h post-transfection, cells were UV-irradiated. After 6 h, cells were lysed and analyzed for luciferase activity. The pRL-TK plasmid was used as an internal control for transfection efficiency.

### Statistics

Statistical significance was performed using the Student' s *t*-test. Results are presented as means±SEM. All P-values quoted are two tailed and significance was accepted when P<0.05.
